# Underpinning the use of indium as a neutron absorbing additive in zirconolite by X-ray absorption spectroscopy

**DOI:** 10.1038/s41598-023-34619-5

**Published:** 2023-06-08

**Authors:** Lewis R. Blackburn, Luke T. Townsend, Malin C. Dixon Wilkins, Toshiaki Ina, Merve Kuman, Shi-Kuan Sun, Amber R. Mason, Laura J. Gardner, Martin C. Stennett, Claire L. Corkhill, Neil C. Hyatt

**Affiliations:** 1 Immobilisation Science Laboratory (ISL), Department of Materials Science and Engineering, University of Sheffield, Sir Robert Hadfield Building, Mappin Street, Sheffield, S13JD UK; 2 Institute of Materials Research, Washington State University, WA 99164 Pullman, USA; 3 Spring8 (JASRI), 1-1-1 Kouto, Sayo-cho, Sayo-gun, Hyogo 679-5198 Japan; 4 School of Materials Science and Energy Engineering, Foshan University, Foshan, 528000 China; 5 School of Earth Sciences, University of Bristol, BS8 1RJ Bristol, UK; 6 School of Mechanical and Materials Engineering, Washington State University, WA 99164 Pullman, USA

**Keywords:** Nuclear waste, Materials science

## Abstract

Indium (In) is a neutron absorbing additive that could feasibly be used to mitigate criticality in ceramic wasteforms containing Pu in the immobilised form, for which zirconolite (nominally CaZrTi_2_O_7_) is a candidate host phase. Herein, the solid solutions Ca_1-x_Zr_1-x_In_2x_Ti_2_O_7_ (0.10 ≤ x ≤ 1.00; air synthesis) and Ca_1-x_U_x_ZrTi_2-2x_In_2x_O_7_ (x = 0.05, 0.10; air and argon synthesis) were investigated by conventional solid state sintering at a temperature of 1350 °C maintained for 20 h, with a view to characterise In^3+^ substitution behaviour in the zirconolite phase across the Ca^2+^, Zr^4+^ and Ti^4+^ sites. When targeting Ca_1-x_Zr_1-x_In_2x_Ti_2_O_7_, single phase zirconolite-2M was formed at In concentrations of 0.10 ≤ x ≤ 0.20; beyond x ≥ 0.20, a number of secondary In-containing phases were stabilised. Zirconolite-2M remained a constituent of the phase assemblage up to a concentration of x = 0.80, albeit at relatively low concentration beyond x ≥ 0.40. It was not possible to synthesise the In_2_Ti_2_O_7_ end member compound using a solid state route. Analysis of the In K-edge XANES spectra in the single phase zirconolite-2M compounds confirmed that the In inventory was speciated as trivalent In^3+^, consistent with targeted oxidation state. However, fitting of the EXAFS region using the zirconolite-2M structural model was consistent with In^3+^ cations accommodated within the Ti^4+^ site, contrary to the targeted substitution scheme. When deploying U as a surrogate for immobilised Pu in the Ca_1-x_U_x_ZrTi_2-2x_In_2x_O_7_ solid solution, it was demonstrated that, for both x = 0.05 and 0.10, In^3+^ was successfully able to stabilise zirconolite-2M when U was distributed predominantly as both U^4+^ and average U^5+^, when synthesised under argon and air, respectively, determined by U L_3_-edge XANES analysis.

## Introduction

Zirconolite (ideally monoclinic CaZrTi_2_O_7_; space group C2/c; Z = 8) is a candidate wasteform material for the immobilisation of actinides arising from reprocessing of spent nuclear fuel (SNF), such as U and Pu^1–4^. The United Kingdom inventory of separated Pu is forecast to reach approximately 140 teHM (tonnes equivalent heavy metal) upon completion of the ongoing reprocessing campaign, which terminated in July 2022. At present, the UK policy favours reuse; material not suitable for this purpose should be immobilised to place beyond reach^5^. Should this policy not prove implementable, the technology to immobilize and dispose of the inventory would be required^6^. At present, the most technically feasible immobilisation pathway would see the conversion of the bulk inventory into solid ceramic monoliths, produced either by a campaign of conventional cold-press sintering (CPS) or hot isostatic pressing (HIP), prior to disposal in a geological disposal facility (GDF)^7,8^.

It may be desirable for wasteforms containing a high fissile content to incorporate a suitable quantity of neutron absorbing additives to mitigate the potential for a criticality event to occur^9,10^. The role of the neutron absorber within the wasteform is to ensure that the probability of a criticality event is mitigated by reducing the internal neutron flux via absorption. Accordingly, during wasteform development and composition scoping trails, during which potential solid solution regimes between the actinide portion and the host material are devised and characterised, it is desirable to ensure that sufficient concentrations of neutron absorbers can be co-accommodated. At present, a range of such additives and their respective incorporation mechanisms for zirconolite have been devised, mainly deploying Gd^3+^ and/or Hf^4+^^2,11–15^. Whilst Gd^3+^ is typically accommodated over the Ca^2+^ and/or Zr^4+^ sites, requiring charge balance via a charge balancing species typically substituted for Ti^4+^ e.g. Ca_1-x_Zr_1-x_Gd_2x_Ti_2_O_7_ or Ca_1-x_Gd_x_ZrTi_2-x_Al_x_O_7_, Hf^4+^ can be directly substituted for Zr^4+^ on the basis of near identical ionic radii (0.78 and 0.76 Å in sevenfold coordination, respectively), resulting in a very minor variation in unit cell volume^16^. However, there are a range of relatively uninvestigated additives that have been proposed as neutron poisons, such as Sm, Dy, Cd, B and In. At present, there is little to no reported data of In, Cd or B substitution within zirconolite and related titanate phases.

The incorporation of In^3+^ within zirconolite was reported by Begg et al., in which In^3+^ was deployed as a Ti^3+^ simulant^17^. A solid solution targeting CaZrTi_2-x_In_x_O_7-2/x_ was fabricated by a cold press and sinter route, with a 1400 °C sintering temperature maintained for 20 h in air, with In^3+^ introduced as In_2_O_3_, targeting x = 0.25, 0.50 and 1.00. It was observed that zirconolite became unstable relative to perovskite and fluorite with increased substitution of In, with a zirconolite yield of 90%, 65% and 0% observed for x = 0.25, 0.50 and 1.00, respectively. It should be noted that In^3+^ substitution in the above solid solution was not charge balanced, with In^3+^ included to simulate the effects of Ti^3+^ ingrowth under reducing conditions rather than as a neutron poison. Another reported example of In-substitution in a zirconolite-like structure was KIn_0.33_Ti_0.67_Te_2_O_7_ fabricated by Lee et al., however this structure was shown to crystallise with orthorhombic unit cell symmetry in the space group Cmcm^18^. This material was reported to form a 3D framework in which each In^3+^ cation was bonded to 6 O atoms in octahedral coordination, again indicating that In^3+^ cations may preferentially occupy the Ti^4+^ sites in the CaZrTi_2_O_7_ structure. Herein, two novel zirconolite systems were investigated, the first of which was Ca_1-x_Zr_1-x_In_2x_Ti_2_O_7_, whereby In^3+^ was targeted across both Ca^2+^ and Zr^4+^ cation sites in equimolar quantity, resulting in a self-charge balancing solid solution. As will later be discussed, the first part of this study demonstrated that In^3+^ preferentially occupied the Ti^4+^ site in zirconolite; hence, the Ca_1-x_U_x_ZrTi_2-2x_In_2x_O_7_ solid solution was also investigated, processed under both Ar and air, with the view to determine whether In^3+^ could successfully charge balance U^4+^ and U^5+^, respectively, at moderately low concentrations of U (x = 0.05 and 0.10).

## Experimental methodology

### Materials synthesis

#### Caution

Uranium is an alpha emitter. Manipulations, synthesis and characterisation was performed in a materials radiochemistry laboratory in a controlled area, using HEPA filtered fume hoods and a dedicated glovebox, following risk assessments and monitoring procedures.

Two solutions targeting Ca_1-x_Zr_1-x_In_2x_Ti_2_O_7_ (0.10 ≤ x ≤ 1.00, Δx = 0.10) and Ca_1-x_U_x_ZrTi_2-2x_In_2x_O_7_ (x = 0.05, 0.10) were fabricated by a conventional mixed oxide synthesis route, whereby the precursors CaTiO_3_ (Sigma Aldrich, 99.9% trace metals basis), ZrO_2_ (Sigma Aldrich, 99.9% trace metals basis), TiO_2_ (anatase, Sigma Aldrich, 99.9% trace metals basis), In_2_O_3_ (Sigma Aldrich, 99.9% trace metals basis) and UO_2_ (ABSCO Ltd., depleted) were intimately mixed by planetary milling. Each reagent was dried at 800 °C prior to weighing, and added to a 45 mL ZrO_2_-lined milling jar with isopropanol and ZrO_2_ milling media. Each sample was homogenised at 400 rpm for 20 min, with the direction of milling reversed after 10 min intervals. The powder slurries were decanted and allowed to dry overnight at 80 °C to evaporate excess solvent; dried powders were further mixed by hand to break up agglomerates and pressed into the walls of a hardened steel die (ø = 10 mm) under 3 tonnes of uniaxial force to form green bodies. These pellets were then placed onto a ZrO_2_ crucible and sintered at 1350 °C for 20 h (air for Ca_1-x_Zr_1-x_In_2x_Ti_2_O_7_; flowing Ar and air for Ca_1-x_U_x_ZrTi_2-2x_In_2x_O_7_).

### Materials characterisation

A portion of each sintered pellet was retained and finely ground for powder X-ray diffraction (XRD) using a Bruker D2 Phaser (Cu Kα source: λ = 1.5418 Å, Ni Filter) fitted with a Lynxeye Position Sensitive Detector, operating at 30 kV and 10 mA. Data were collected in the range 10° ≤ 2θ ≤ 70° with a stepsize of 0.02° s^−1^. Phase identification and peak indexing was achieved using the PDF4+ database and Rietveld analysis was performed using the Bruker TOPAS package. Scanning electron microscopy (SEM) analysis was performed using a Hitachi TM3030 operating with a 15 kV accelerating voltage at a working distance of 8 mm, fitted with a Bruker Quantax 70 spectrometer for Energy Dispersive X-ray Spectrometry (EDS). Samples were prepared for SEM–EDS analysis by mounting in cold setting epoxy resin and curing for 24 h, prior to grinding using incremental grades of SiC paper, before polishing to a 1 μm optical finish using diamond suspension. In K-edge X-ray absorption spectroscopy (XAS) data were acquired at BL01B1, SPring-8 (Hyogo, Japan). The storage ring energy was operated at 8 GeV with a typical current of 100 mA. Measurements were obtained using a Si (311) double-crystal monochromator in transmission mode at room temperature. Data were collected alongside In_2_O_3_ and In foil reference compounds. U L_3_-edge fluorescence and Zr K-edge transmission XAS data were collected at Diamond Light Source Beamline B18 (Oxford, UK), alongside a selection of reference compounds, containing U and Zr in a variety of coordination environments and U-oxidation states. A Si(111) double crystal monochromator was used to fine-tune incident synchrotron radiation, with the intensity of the incident and transmitted beam measured using ionization chambers, filled with a mixture of N_2_ and He gas, operated in a stable region of the I/V curve. Data reduction and analysis was achieved using the Demeter software package^19^. Bond valence sums were calculated using the in-built function in the Artemis software package. Here, the bond valence sum parameters are derived from the work of Altermatt and Brown^20,21^ and use the following equation:

$${s}_{ij}={e}^{\left[\frac{{R}_{ij}-{R}{^{\prime}}_{ij}}{b}\right]}$$

where s_ij_ is the bond valence, R_ij_ is the bond distance between atoms i and j and R′_ij_ and b are the empirically determined parameters by Altermatt and Brown.

## Results and discussion

### Phase evolution in the Ca_1-x_Zr_1-x_In_2x_Ti_2_O_7_ system

**Figure 1:**
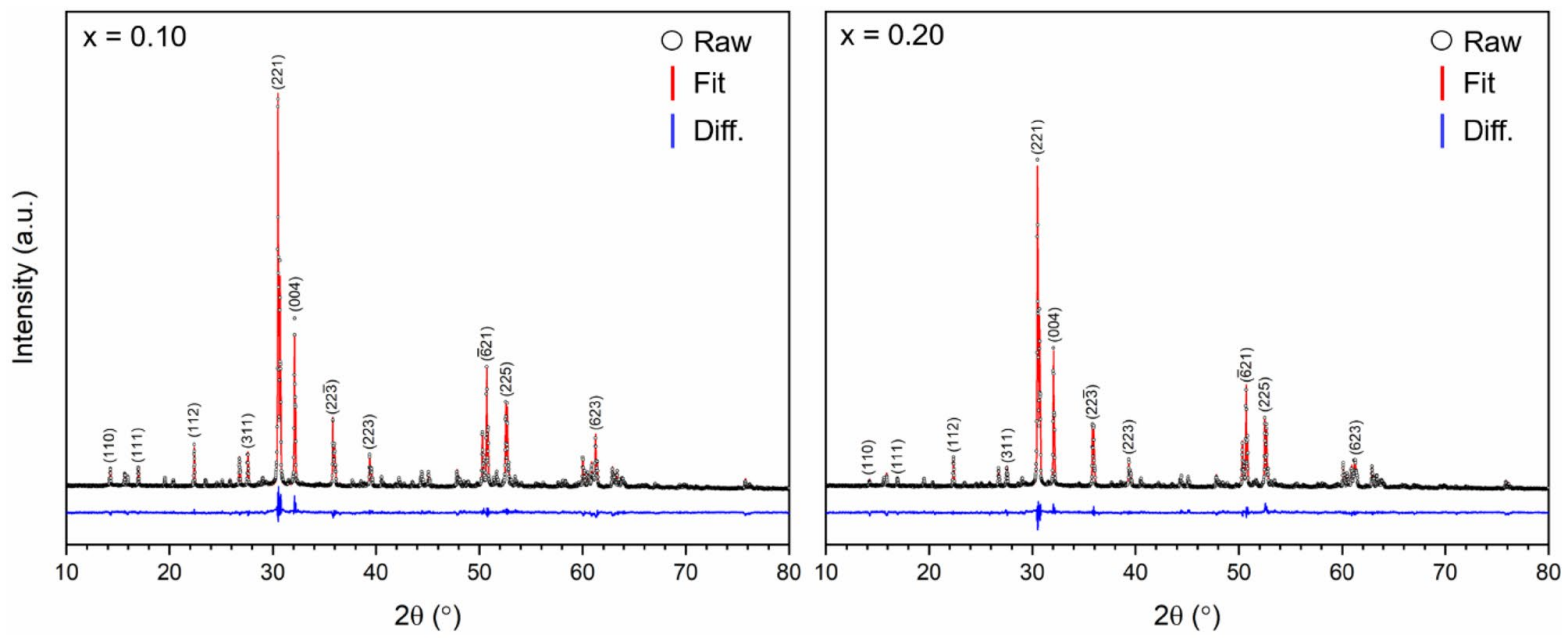
Rietveld refinement data for x = 0.10 (left) and x = 0.20 (right) compositions in the Ca_1-x_Zr_1-x_In_2x_Ti_2_O_7_ system fit to the zirconolite-2M structural model.

**Figure 2:**
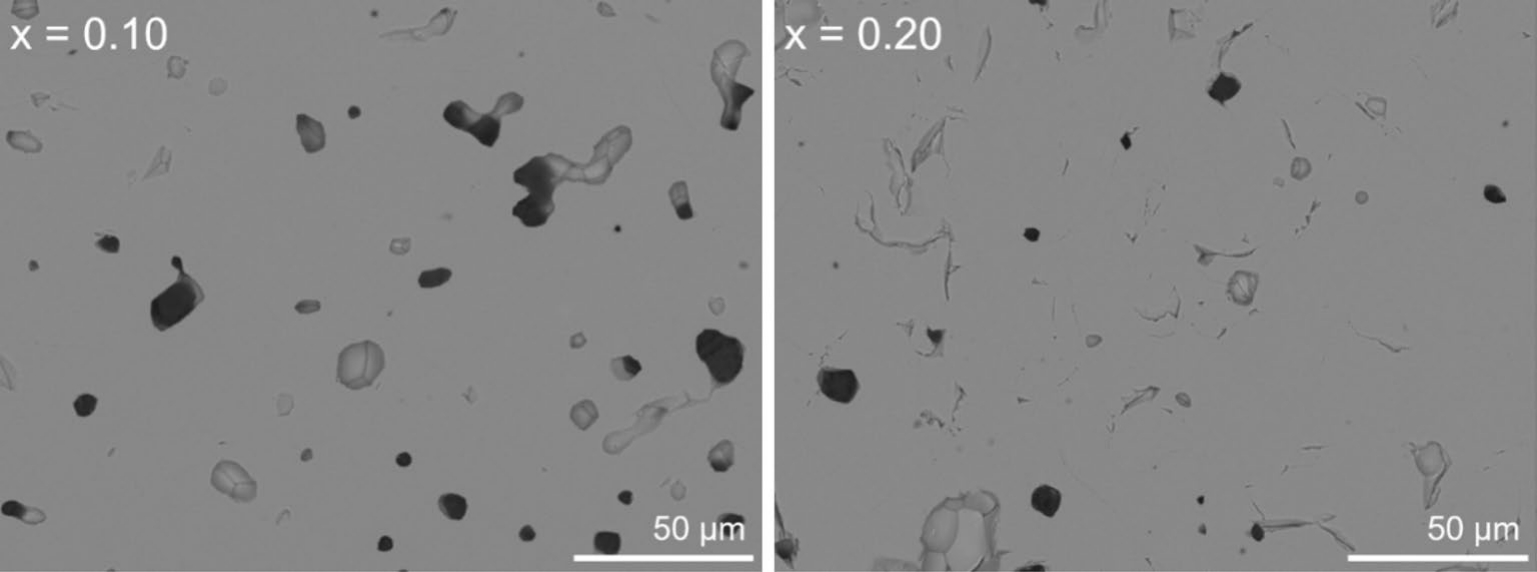
BSE images for x = 0.10 (left) and x = 0.20 (right) in the Ca_1-x_Zr_1-x_In_2x_Ti_2_O_7_ solid solution.

Powder XRD data for the formulations x = 0.10 and 0.20 were consistent with the formation of single phase zirconolite-2M. Unit cell dimensions were obtained by Rietveld analysis of powder diffraction data (Fig. 1, Table 1) corresponding to a decrease in the unit cell volume with increased In content. The unit cell parameters of pure CaZrTi_2_O_7_ have been previously reported as: a = 12.4462 Å, b = 7.2717 Å, c = 11.3813 Å, β = 100.5587° and V = 1012.60 Å^3^^22^. There was therefore a consistent reduction in the lattice constants of the zirconolite-2M with respect to In substitution. No reflections representative of common secondary phases (ZrO_2_, CaTiO_3_) were evidenced in the diffraction patterns. Moreover, combined SEM–EDS analyses indicate the formation of high density microstructures comprised of a matrix of zirconolite-2M, with no phase separation visible by variation in backscattered electron contrast (Fig. 2). EDS analysis of the zirconolite-2M matrix formed for x = 0.10 and 0.20 confirmed the presence of In, indicated by the prominence of the In Lα and In Lβ emission lines present in the EDS spectrum (Fig. S1). The average composition of the zirconolite phase(s) was determined by semi-quantitative EDS analysis, normalised to seven oxygen atoms; the observed compositions are in good agreement with the targeted formulation (Table S1).

**Table 1:** Unit cell parameters for single phase In-doped zirconolite-2M compositions (x = 0.10, 0.20) in the Ca_1-x_Zr_1-x_In_2x_Ti_2_O_7_ system.

Composition	Unit cell parameters					R_wp_ (%)	χ^2^
	a (Å)	b (Å)	c (Å)	β (˚)	V (Å^3^)		
Ca_0.90_Zr_0.90_In_0.20_Ti_2_O_7_	12.45215 (14)	7.26300 (8)	11.36556 (14)	100.512 (1)	1010.65 (2)	8.96	1.77
Ca_0.80_Zr_0.80_In_0.40_Ti_2_O_7_	12.44757 (16)	7.24993 (9)	11.36477 (14)	100.441 (1)	1008.62 (2)	8.66	1.61

**Figure 3:**
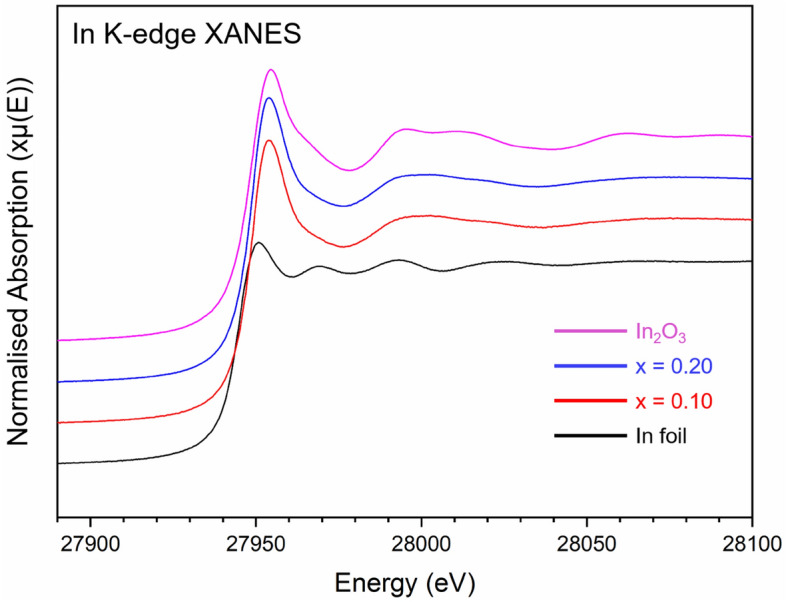
In K-edge XANES spectra for In-doped zirconolite-2M at concentrations x = 0.10 and 0.20, alongside In_2_O_3_ and In foil reference compounds.

The In K-edge XANES spectra for single phase zirconolite-2M (x = 0.10 and 0.20) compositions are presented in Fig. 3. Data were collected alongside In_2_O_3_ and In foil reference compounds, representing In^3+^ and In^0^ oxidation states, respectively. For both In-containing zirconolite-2M compositions, the XANES spectra when overlaid were practically indistinguishable, indicative of identical speciation and coordination of In cations when targeting both x = 0.10 and 0.20. These spectra were comprised of a single intense absorption feature (E_0_ = 27,945.5 eV) followed by a weak post-edge oscillation, with maxima at 28,000 eV. It was clear that position of the absorption edge of x = 0.10 and 0.20 compounds was identical to that of the In_2_O_3_ reference compound (space group Ia$\overline{3 }$) which contains trivalent In^3+^ distributed across two sites, both of which are sixfold coordinated to O^2−^, inferring uniform In^3+^ speciation within the zirconolite-2M phase. Moreover, linear combination fitting of the XANES spectra for x = 0.10 and 0.20 confirmed 100% In^3+^ speciation relative to In_2_O_3_ and In-foil reference compounds, with R-factors of 0.00469 and 0.00531, respectively.

**Figure 4:**
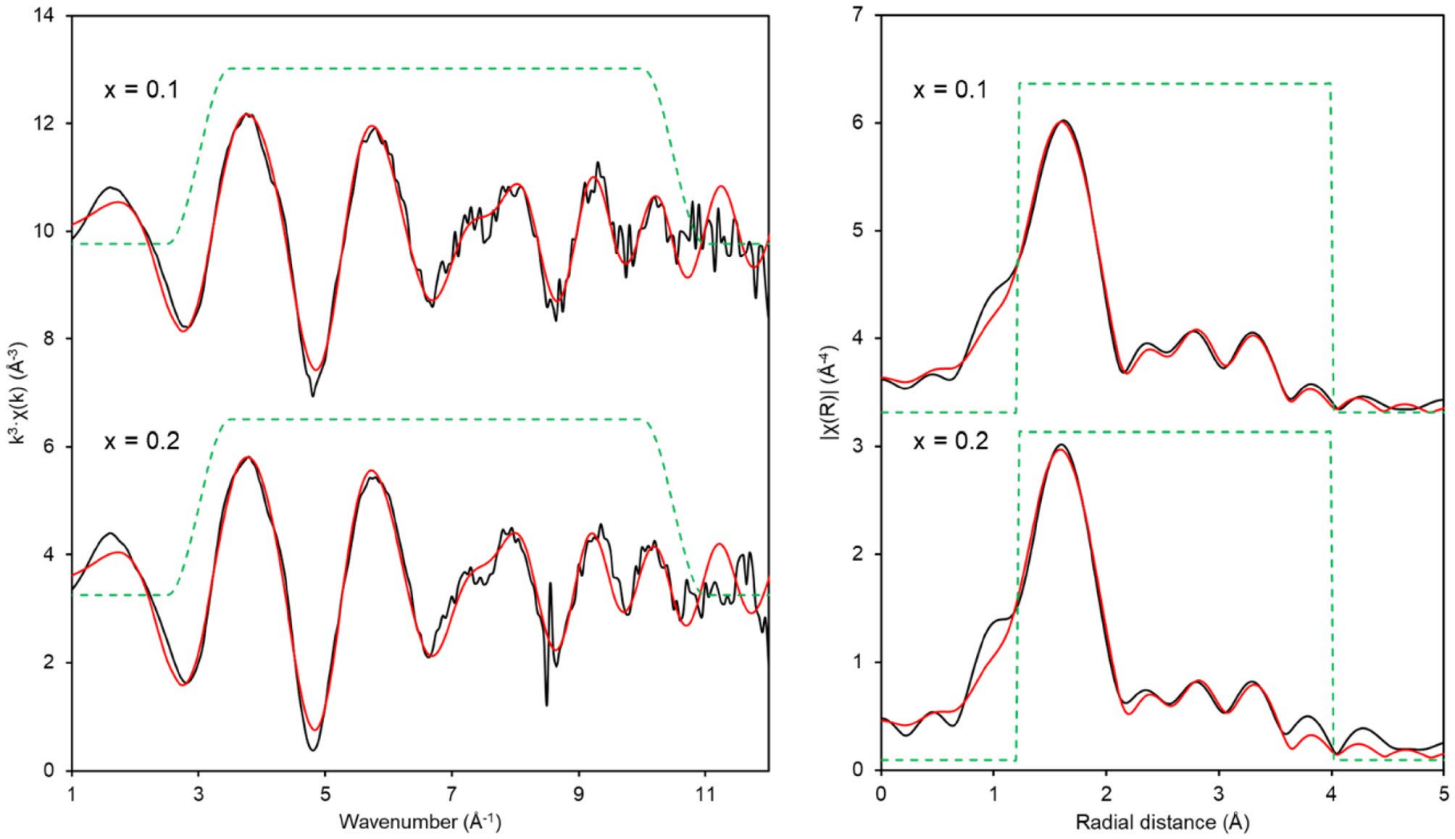
In K-edge XAS spectra for x = 0.10 and 0.20 compositions in the Ca_1-x_Zr_1-x_In_2x_Ti_2_O_7_ system. Left—*k*^3^-weighted EXAFS. Right—Fourier transform of the *k*^3^-weighted EXAFS, using a Hanning window function. Black lines are data and red lines are the best fit models for the data.

Fitting of the EXAFS spectra (Fig. 4, Table 2) for both the x = 0.10 and 0.20 compositions produced good fits and utilised very similar models that corresponded well to the expected zirconolite-2M structure. For x = 0.10, the best fit model (R-factor = 0.0078) included 6 O backscatterers at 2.16(1) Å, 2 Ti backscatterers at 3.33(1) Å, and 2 Ti backscatterers at 3.57(1) Å. For x = 0.20, the best fit model (R-factor = 0.0106) included a split first O shell, with 3 O backscatterers at 2.11(6) Å and 3 O backscatterers at 2.21(6) Å, 2 Ti backscatterers at 3.34(2) Å, and 2 Ti backscatterers at 3.58(2) Å. Both fits match well with the expected structure of zirconolite-2M according to Whittle et al.^23^, with the exception of the second Ti shell at ~ 3.58 Å exhibiting an increase in the expected degeneracy (2 instead of the expected 1) since the Ca backscatterer at the same interatomic distance (R_eff_ = 3.57 Å) was not modelled to avoid over-parameterising the model. Given both the second Ti shell and the Ca backscatterers manifest at very similar areas of the EXAFS spectrum, it is likely that significant interference makes fitting the expected shells and their corresponding degeneracies (i.e. 1 Ti and 1 Ca as opposed to the best fit model 2 Ti) challenging. Similar challenges in fitting have been observed in other doped zirconolite systems^24^ whereby the delineation of the exact type and number of backscatterers in the approximate range 3.5–3.6 Å was found to be not possible.

**Table 2:** Fitting parameters for In K-edge EXAFS data presented in Fig. 4.

Sample	Parameters	Path			
		O1	O2	Ti1	Ti2
x = 0.10	N	6	–	2	2
E_0_ = 0.7(10)	σ^2^ (10^−3^) (Å^2^)	16 (1)	–	8 (2)	7 (2)
R-factor = 0.0079	R (Å)	2.16 (1)	–	3.33 (1)	3.58 (2)
BVS = 2.97	α (%)	100.0	–	100.0	100.0

x = 0.20	N	3	3	2	2
E_0_ = 0.3(14)	σ^2^ (10^−3^) (Å^2^)	10 (5)*	10 (5)*	8 (2)	7 (3)
R-factor = 0.0117	R (Å)	2.10 (3)	2.23(3)	3.34 (2)	3.59 (2)
BVS = 3.01	α (%)	100.0	100.0	100.0	100.0

The amplitude reduction factor (S_0_^2^ = 0.96) for all samples was determined by fitting of the In_2_O_3_ reference compound; N is the degeneracy; σ^2^ is the Debye–Waller factor; R is the interatomic distance; α is the result of the F-test indicating the confidence that adding the path improves the fit (> 67% is equal to 1σ and > 95% is equal to 2σ in terms of standard deviation); BVS is the bond valence sum; *indicates that the parameters were constrained to be equal.

The total degeneracy for the first O shell was found to be six in both samples (6 O at 2.16(1) Å for x = 0.10 and a split O shell of 3 O at 2.11(6) Å and 3 O at 2.21(6) Å for x = 0.20). The sixfold coordination of In^3+^, determined by these EXAFS analyses, implies that some compositional rearrangement is required to maintain charge balance across the single phase zirconolite-2M samples, for example (Ca_1-x_Ti_x_)(Zr_1-x_Ti_x_)Ti_2-2x_In_2x_O_7_, consistent with the known defect mechanisms in non-stoichiometric zirconolite^25^. Indeed, the only previous study that explored In doping of zirconolite found that In readily expresses a preference for the fivefold Ti site^17^. The total degeneracy, and by extension the inferred mixture of In^3+^ coordination environments, correspond well to the bond valence sums (BVS) which sum to approximately 3 (2.973 for x = 0.10 and 3.014 for x = 0.20) for both samples. When trialing other models, with In^3+^ solely on the alternative Ti site (total O degeneracy of 5), the Ca site (total O degeneracy of 8) or Zr site (total O degeneracy of 7) the calculated BVS diverged markedly from the expected value of 3, and the overall model produced a qualitatively and quantitatively worse fit to the data.

**Figure 5:**
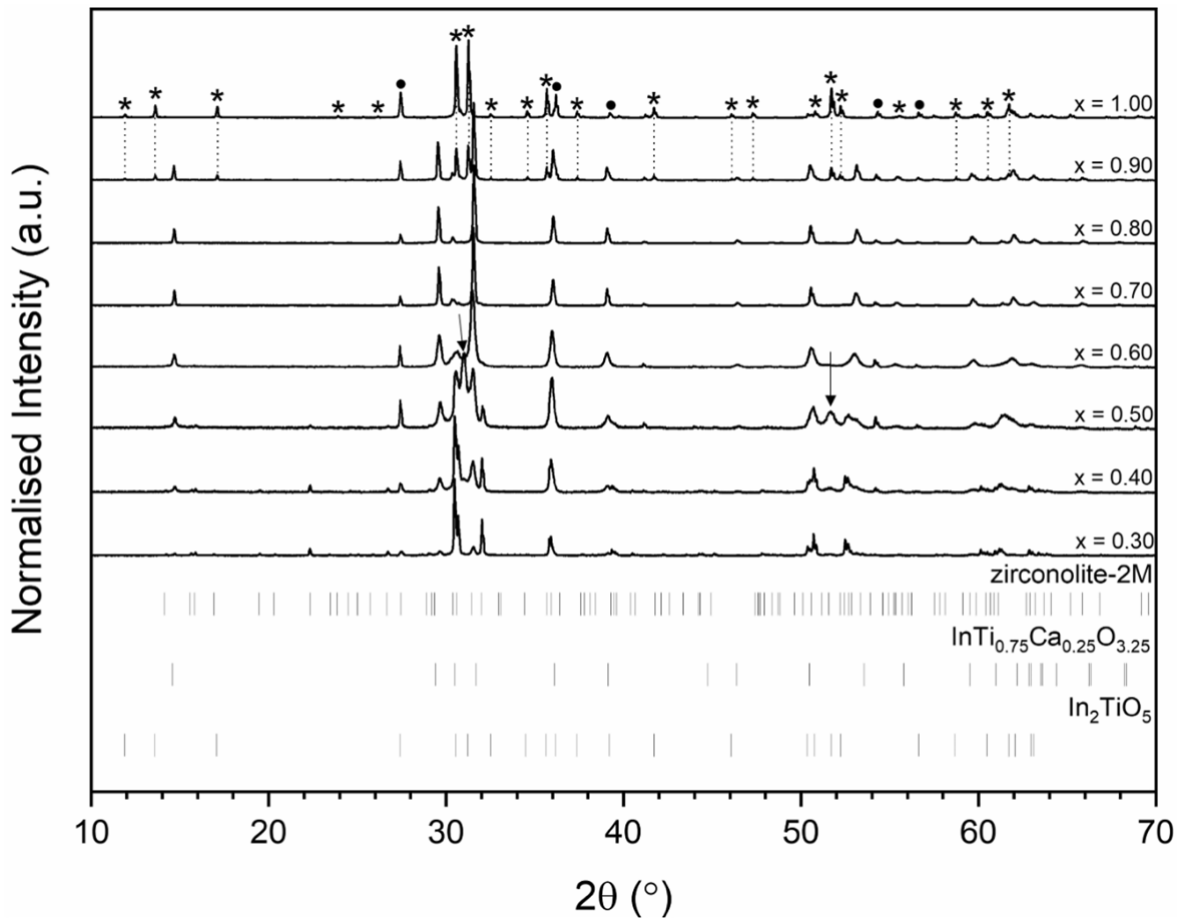
Powder XRD data for 0.30 ≤ x ≤ 1.00 compositions in the Ca_1-x_Zr_1-x_In_2x_Ti_2_O_7_ system. TiO_2_ reflections are labelled with closed circles. In_2_TiO_5_ reflections are labelled with stars. The prominent reflections contributing to In-doped c-ZrO_2_ are indicated with arrows.

**Figure 6:**
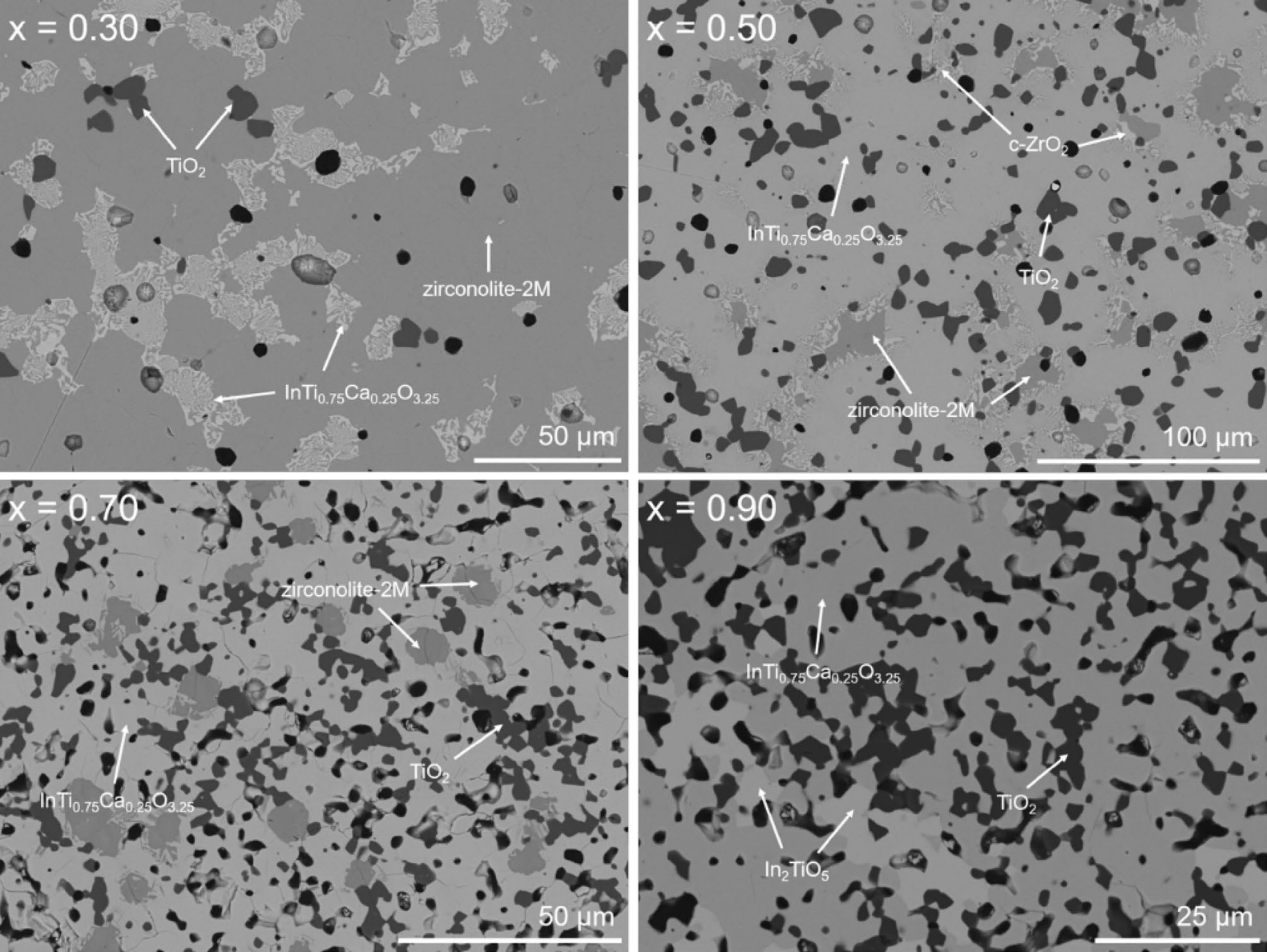
SEM analysis of x = 0.30, 0.50, 0.70 and 0.90 compositions in the Ca_1-x_Zr_1-x_In_2x_Ti_2_O_7_ solid solution.

As the targeted In^3+^ concentration was progressed beyond x ≥ 0.20, zirconolite-2M was no longer isolated as a single phase, with a number of ancillary In-bearing phases clearly distinguished by both XRD and combined SEM–EDS analyses. Nevertheless, zirconolite was detected by XRD up to x = 0.80, albeit at very low concentration (approximately 3 wt% at x = 0.80; see quantitative phase analysis in Table 3). Figure 5 shows the powder XRD data for compositions in the range 0.30 ≤ x ≤ 1.00. When targeting x = 0.30, a number of additional reflections corresponding to TiO_2_ (P4_2_/mnm) and InTi_0.75_Ca_0.25_O_3.25_ (C2/m) were clearly observed, accounting for 2.3 ± 0.3 and 12.9 ± 0.3 wt% of the overall phase assemblage, respectively. SEM analysis (Fig. 6) confirmed the presence of all phases identified by XRD in the microstructure. In the compositional interval 0.40 ≤ x ≤ 0.60, an additional In-bearing cubic ZrO_2_ phase was also observed (highlighted by arrows in Fig. 5), plateauing with a maximum fraction of ~ 24 wt% of the overall phase assemblage at x = 0.50. The relative concentrations of InTi_0.75_Ca_0.25_O_3.25_ and TiO_2_ steadily increased, with a corresponding decrease in the zirconolite-2M phase. It was interesting to note that zirconolite did not appear to undergo any structural transformations to the 4M or 3T polytypes, as is typically observed with excessive doping in the Ca^2+^ and/or Zr^4+^ sites^1^. When targeting the end-member In_2_Ti_2_O_7_ composition (i.e. x = 1.00), a two phase mixture of In_2_TiO_5_ (79.4 ± 0.34 wt%) and TiO_2_ (20.6 ± 0.34 wt%) was identified by XRD and confirmed by SEM–EDS analyses, consistent with previous data^26^. The crystal structure of the cubic pyrochlore phase is characterized as an anion-deficient fluorite superstructure, adopting the generic A_2_B_2_O_7_ stoichiometry, resulting in two distinct cation sites; A cations are typically larger trivalent atoms (e.g. REE^3+^ = Dy, Y, Sm, Gd) whereas the B site is comprised of smaller, higher valence cations such as Ti^4+^ and Hf^4+^, although a number of variations do exist^27^. The stability of the cubic pyrochlore phase (space group Fd $\overline{3 }$ m) is dictated by the size ratio of r_A_/r_B_ whereby the A and B sites are eight and sixfold coordinated to O^2−^, respectively. Varying A and B site cations such that r_A_/r_B_ > 1.78 results in the formation of a monoclinic structure, whereas r_A_/r_B_ < 1.46 promotes the defect-fluorite structure type, in which oxygen vacancies are disordered across the sub-lattice. Considering the respective ionic radii of In^3+^ and Ti^4+^ in eight and sixfold coordination (0.92 Å and 0.605 Å) and the corresponding ratio (r_A_/r_B_ = 1.52) the In_2_Ti_2_O_7_ phase should form the cubic pyrochlore structure on the basis of ionic radius ratio, nevertheless, no yield of In_2_Ti_2_O_7_ was observed at 1350 °C. Despite further attempts to form this phase with an increased sintering temperature of 1700 °C and extended dwell time of 24 h, we noted that these conditions were sufficient to completely volatilise the pellet during sintering and no yield was obtained.

**Table 3:** Quantitative phase analysis (QPA) for the Ca_1-x_Zr_1-x_In_2x_Ti_2_O_7_ solid solution calculated from Rietveld analysis of powder XRD data (* indicates phase purity).

Composition	Phase assemblage (wt%)					R_wp_ (%)	χ^2^
	Zirconolite-2M	InTi_0.75_Ca_0.25_O_3.25_	TiO_2_	In-doped c-ZrO_2_	In_2_TiO_5_		
x = 0.10	100*	–	–	–	–	8.96	1.77
x = 0.20	100*	–	–	–	–	8.66	1.61
x = 0.30	84.8 ± 0.3	12.9 ± 0.3	2.3 ± 0.3	–	–	8.48	1.55
x = 0.40	57.5 ± 0.6	24.1 ± 0.5	5.8 ± 0.2	12.6 ± 0.7	–	8.70	1.57
x = 0.50	32.3 ± 0.4	35.5 ± 0.5	8.4 ± 0.2	23.9 ± 0.4	–	8.23	1.51
x = 0.60	17.7 ± 1.1	66.9 ± 1.6	10.2 ± 0.5	5.2 ± 0.7	–	9.08	1.59
x = 0.70	8.9 ± 0.4	81.9 ± 0.7	9.2 ± 0.4	–	–	11.83	2.12
x = 0.80	3.0 ± 0.2	89.4 ± 0.5	7.6 ± 0.3	–	–	11.86	2.17
x = 0.90	–	63.4 ± 1.3	13.6 ± 0.5	–	23.0 ± 0.8	10.79	1.87
x = 1.00	–	–	20.6 ± 0.3	–	79.4 ± 0.3	9.78	1.69

### U L_3_ and Zr K-edge X-ray absorption near edge structure (XANES) analysis of the Ca_1-x_U_x_ZrTi_2-2x_In_2x_O_7_ system

**Figure 7:**
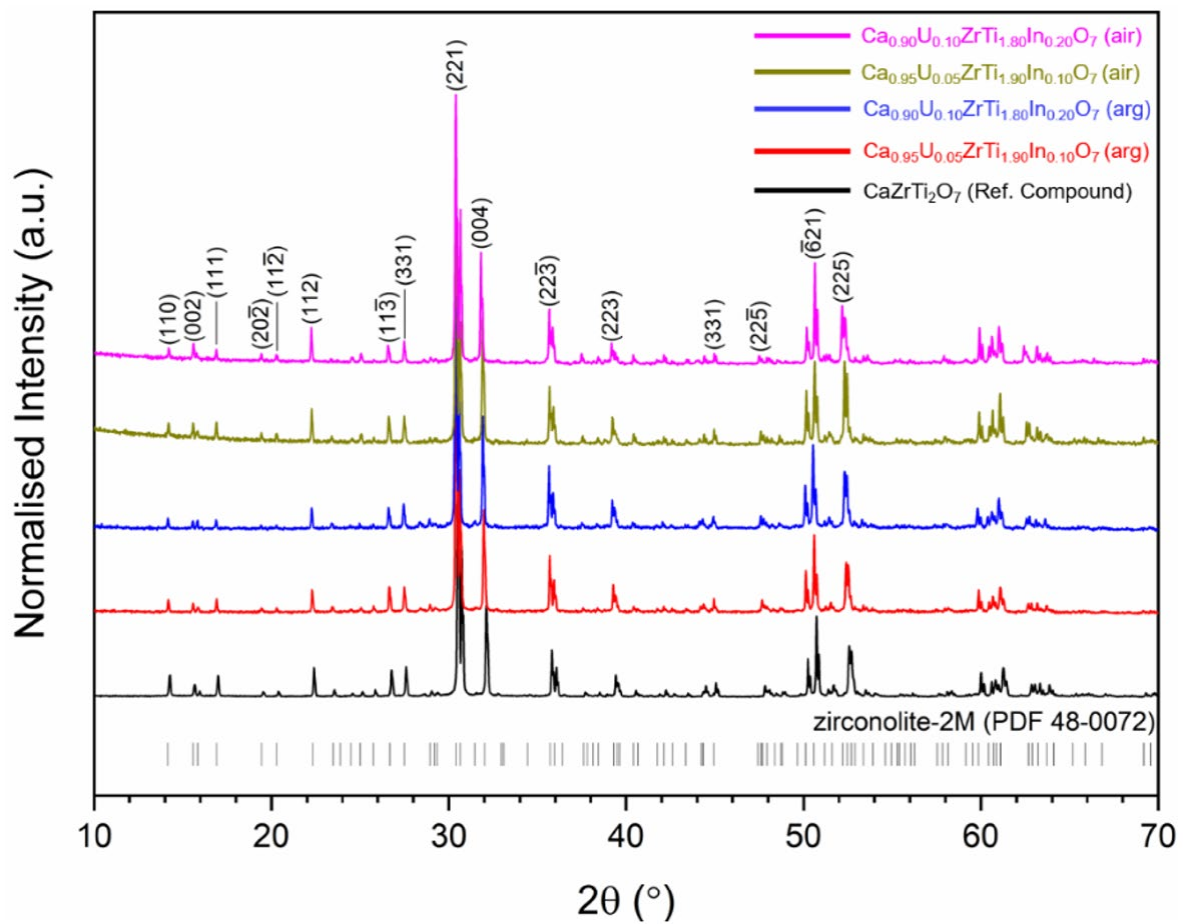
Powder XRD data for Ca_0.95_U_0.05_ZrTi_1.90_In_0.10_O_7_ (x = 0.05) and Ca_0.90_U_0.10_ZrTi_1.80_In_0.20_O_7_ (x = 0.10) synthesised under both air and argon.

As In adopted the In^3+^ oxidation state when substituted in the Ca_1-x_Zr_1-x_In_2x_Ti_2_O_7_ solid solution, preferentially occupying the Ti site, it may be feasible for In^3+^ to act as a neutron poison and charge balancing species within the Ti^4+^ site. Moreover, to our knowledge, the only other reported instance of In^3+^ substitution in zirconolite was consistent with occupation in the Ti^4+^ site as a simulant for the reduced Ti^3+^ species^17^. Hence, two compositions in the Ca_1-x_U_x_ZrTi_2-2x_In_2x_O_7_ system targeting x = 0.05 and 0.10 were also fabricated, with U deployed as a surrogate for Pu. Samples were first synthesised under argon, with a view to maintain the preferred U^4+^ valence configuration, as this is generally the target oxidation state for immobilised Pu in zirconolite. Powder XRD data for both compositions sintered under flowing Ar gas are shown in Fig. 7, and are consistent with the formation of near single phase zirconolite-2M (a small fraction of ZrO_2_ was also observed) at both targeted concentrations of U, indicating the In^3+^ was successfully accommodated in solid solution. The unit cell parameters of the zirconolite-2M phase(s) were calculated by Rietveld analysis of powder XRD data and are listed in Table 3. A yield of near single phase zirconolite-2M (< 2 wt% free ZrO_2_) was also obtained when compositions were synthesised in air, indicating that In^3+^ may capable of charge balancing U valence states greater than U^4+^.

**Figure 8:**
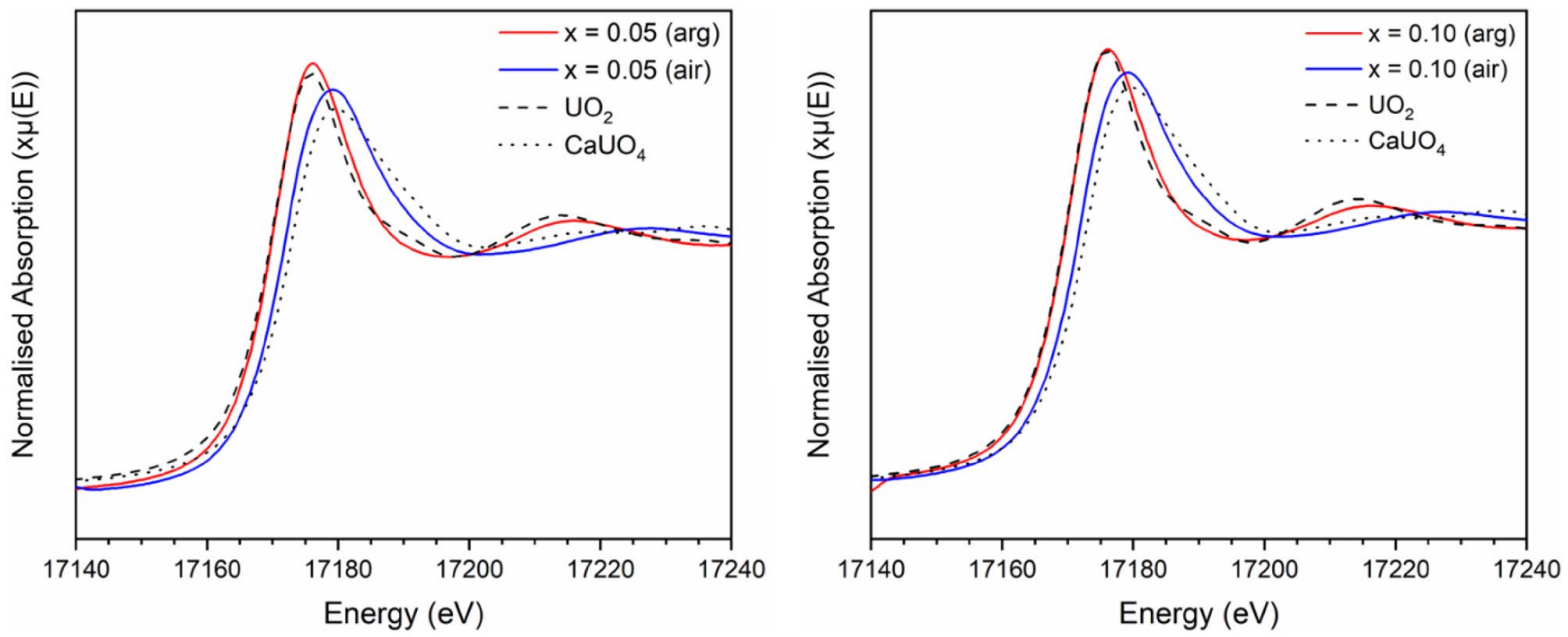
Normalised U L_3_-edge XANES for x = 0.05 (left) and x = 0.10 (right) compositions in the Ca_1-x_U_x_ZrTi_2-2x_In_2x_O_7_ solid solution sintered in argon and air. Data are displayed alongside UO_2_ (dashed) and CaUO_4_ (dotted) reference compounds, representing U^4+^ and U^6+^, respectively.

In order to determine the formal oxidation state of U, X-ray absorption near edge structure (XANES) spectra were collected at the U L_3_-edge (~ 17,166 eV) in transmission mode (Fig. 8) alongside a series of reference compounds containing U with known oxidation state and coordination environment. Scanning across the U L_3_ absorption edge probes the dipole transition from a core 2p_3/2_ shell to the partially occupied 6d valence shell. The observed spectra for all measured compounds were comprised of a single intense absorption feature with additional resonance features present above the main absorption edge; the energy position of the absorption edge is correlated to the formal oxidation state of the absorbing atom in many actinide species.

**Figure 9:**
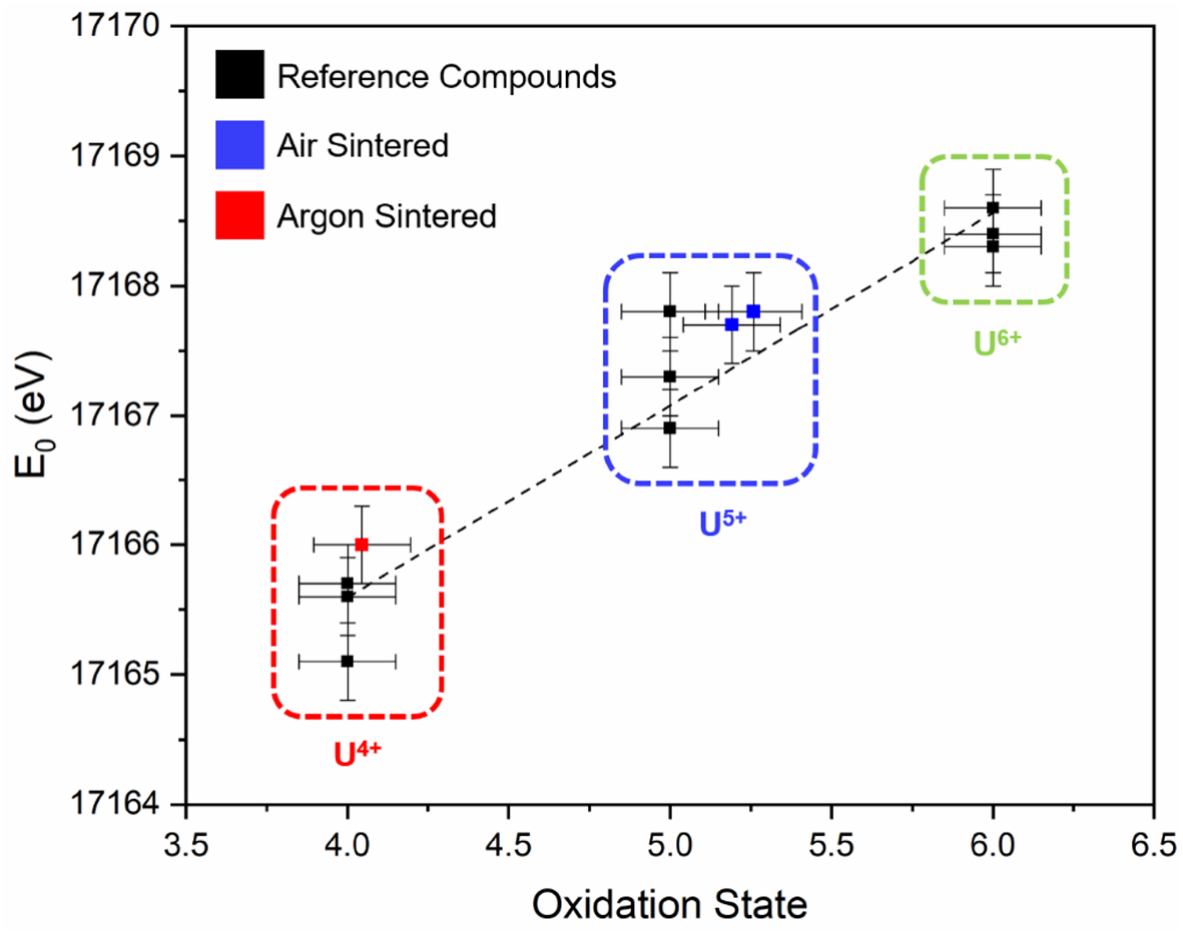
Oxidation state of U in Ca_1-x_U_x_ZrTi_2-2x_In_2x_O_7_ (x = 0.05, 0.10; air and argon) calculated by linear regression of E_0_ position relative to reference compounds (U^4+^O_2_, U^4+^Ti_2_O_6_, U^4+^SiO_4_, U^5+^SbO_5_, U^5+^MoO_4_, LaU^5+^O_5_, CaU^6+^O_4_, Ca_3_U^6+^O_6_ and MgU^6+^O_4_).

The E_0_ position of compositions sintered under flowing Ar was measured as the energy for the normalised absorbance μx = 0.5 (i.e. the energy at half the absorption edge), and was found to be 17,166.0(5) eV for both x = 0.05 and 0.10 compounds, indicative of uniform U speciation at both concentrations. These spectra displayed a clear resemblance to the UO_2_ reference compound with respect to the normalised edge intensity, position and post-edge oscillation feature, consistent with U^4+^ speciation in the zirconolite-2M compounds. The E_0_ position of the U L_3_-edge in the air-synthesised compounds was shifted to higher energy relative to the UO_2_ reference compound, measured to be 17,167.7(5) eV and 17,167.8(5) eV for x = 0.05 and 0.10, respectively, indicative of oxidised U. To establish the average oxidation states of U in both sets of compounds, a linear regression plot of the E_0_ position (defined as μx = 0.5) relative to a large suite of reference compounds was constructed (Fig. 9). The oxidation states of U in the x = 0.05 and 0.10 were calculated to be 4.1 and 4.1 in argon; 5.2 and 5.3 in air. These data are strong evidence to support the use of zirconolite as a potential wasteform for U/Pu, as the zirconolite-2M structure is clearly capable of accommodating changes in oxidation state without phase separation. Moreover, it is clear that In^3+^ is capable of charge balancing both U^4+^ and U^5+^ within the zirconolite structure, in the case of the latter, implying a charge balancing mechanism consistent with Ca_1-x_U^5+^_x_ZrTi_2-2x_In_2x_O_7+x_ (Table 4).

**Table 4:** Unit cell parameters for single phase U-doped zirconolite-2M compositions (x = 0.05, 0.10) in the Ca_1-x_U_x_ZrTi_2-2x_In_2x_O_7_ system, synthesised in both air and argon.

Composition	Atmosphere	Unit cell parameters					R_wp_ (%)	χ^2^
		a (Å)	b (Å)	c (Å)	β (˚)	V (Å^3^)		
Ca_0.95_U_0.05_ZrTi_1.90_In_0.10_O_7_	Air	12.45370 (18)	7.27650 (11)	11.41709 (18)	100.480 (1)	1017.35 (3)	8.07	1.21
Ca_0.950_U_0.10_ZrTi_1.80_In_0.20_O_7_	Air	12.45856 (19)	7.27675 (12)	11.45444 (19)	100.398 (1)	1021.38 (3)	8.80	1.39
Ca_0.95_U_0.05_ZrTi_1.90_In_0.10_O_7_	Argon	12.46110 (20)	7.28045 (12)	11.39714 (18)	100.560 (1)	1016.47 (3)	9.33	1.25
Ca_0.950_U_0.10_ZrTi_1.80_In_0.20_O_7_	Argon	12.47578 (26)	7.28496 (14)	11.41418 (23)	100.544 (2)	1019.87 (4)	9.90	1.25

**Figure 10:**
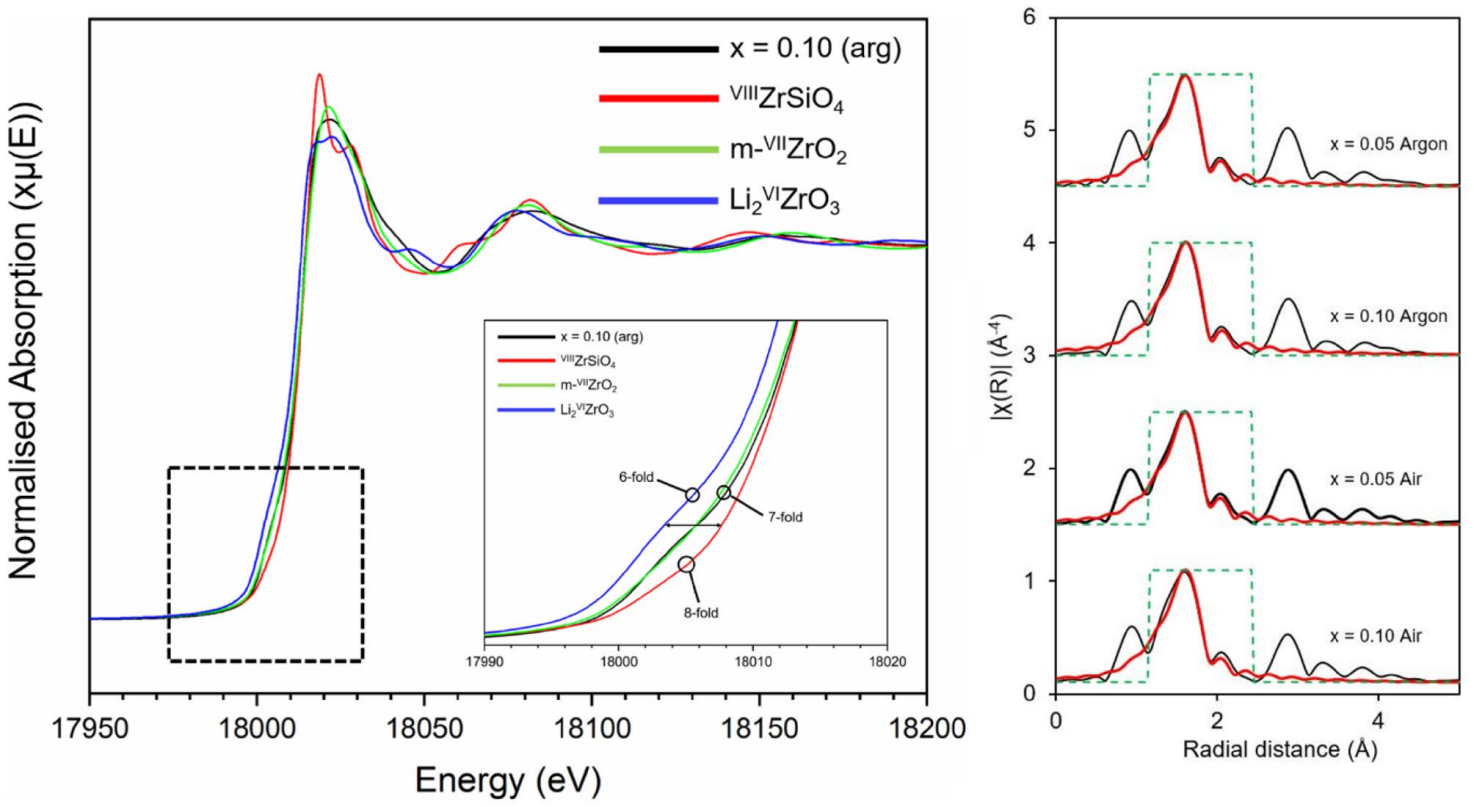
Zr K-edge XAS spectra for Ca_1-x_U_x_ZrTi_2-2x_In_2x_O_7_ (x = 0.05, 0.10) processed under argon and air. Left - Zr K-edge XANES data. Right - *k*^3^-weighted EXAFS of the Zr K-edge using a Hanning function window. The fit shown is for the first coordination shell only; black lines are data and red lines are the best fit models for the data.

**Figure 11:**
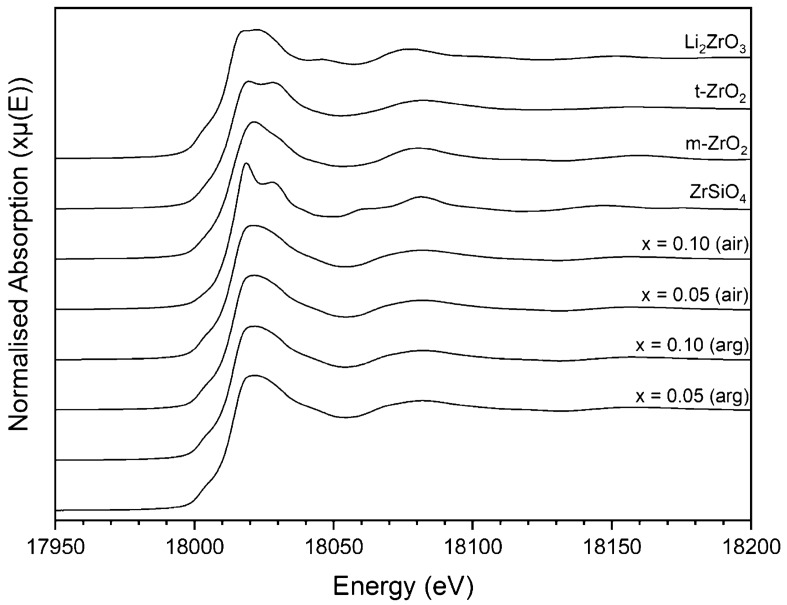
Zr K-edge XANES data for Ca_1-x_U_x_ZrTi_2-2x_In_2x_O_7_ (x = 0.05, 0.10) processed under argon and air. Data are presented alongside a series of Zr reference compounds.

XAS spectra were acquired at the Zr K-edge for x = 0.05 and 0.10 in the Ca_1-x_U_x_ZrTi_2-2x_In_2x_O_7_ solid solution (for both air and argon) alongside a variety of reference compounds containing Zr in distinctive coordination environments (Figs. 10, 11). There were notable differences in the XANES spectra of Zr-containing compounds depending on the coordination of Zr atoms. However, the absorption features for each measured zirconolite compound were observed to be near-identical regardless of targeted U concentration or processing environment, and, in excellent agreement with those previously reported in the literature, including synthetic CaZrTi_2_O_7_^22,28^ and annealed metamict (Ca,Th)ZrTi_2_O_7_ zirconolite^29,30^. The XANES spectra are presented alongside a series of reference compounds, with variation in pre-edge intensity and main white-line absorption features consistent with Zr in a variety of coordination environments^31^. It was observed that the zirconolite and m-ZrO_2_ compounds exhibited rounded features at the crest of the white line, which is typical of sevenfold coordinate Zr, whereas the ZrSiO_4_, t-ZrO_2_ and Li_2_ZrO_3_ compounds exhibited notable peak splitting which is typical of octahedral coordination, or, as in the case of ZrSiO_4_, highly distorted eightfold coordination^22^. Qualitative analysis of the XANES region was consistent with the Zr atoms in zirconolite occupying the expected sevenfold coordination environment, as there are clear parallels with the m-^VII^ZrO_2_ reference compound. The Zr-K edge *k*^3^-weighted EXAFS modelling of the first oxygen coordination shell for each zirconolite specimen is presented in Fig. 10 (Table S2). The same model could be fit to all samples for both atmospheres (x = 0.05 and 0.10 for both argon and air) and was consistent with 7 O backscatterers present around the Zr central atom. In all fits, the first oxygen coordination shell was split into 4 O backscatterers at 2.10–2.11 Å and 3 O at 2.24–2.25 Å. These fits are in good agreement with the expected Zr coordination environment for zirconolite, in which a spread of 7 O backscatterers are present from 2.045 to 2.339 Å^23^. A distinct yet low intensity pre-edge feature was observed for the zirconolite materials and Zr compounds, attributed to the formally forbidden 1s→4d transition; the relative prominence of this feature is increased for compounds for which Zr is located in a distorted, non-centrosymmetric coordination environment. The inset of Fig. 10 highlights the pre-edge region of zirconolite (represented by the x = 0.10 (argon) composition), consistent with sevenfold coordination, alongside ZrSiO_4_, m-ZrO_2_ and Li_2_ZrO_3_, representing eight, seven and sixfold coordinate Zr, respectively. There is a clear trend in the intensity of the pre-edge feature with reduced coordination, exhibited most clearly by the difference between Li_2_^VI^ZrO_3_ and ^VIII^ZrSiO_4_. The increased intensity of this feature is attributed to a lack of centrosymmetry around the absorbing Zr atom. Furthermore, the pre-edge shape and intensity of the zirconolite and m-ZrO_2_ appears similar, further supporting the existence of sevenfold Zr in the U/In-doped zirconolite materials.

## Conclusions

Herein, we have presented a novel examination of the solid solution behaviour of indium within the zirconolite phase, as a potential neutron absorbing additive. Two distinct substitution regimes were devised, targeting Ca_1-x_Zr_1-x_In_2x_Ti_2_O_7_ (targeting In^3+^ distributed equimolar across Ca^2+^ and Zr^4+^ sites) and Ca_1-x_U_x_ZrTi_2-2x_In_2x_O_7_ (targeting In^3+^ across the Ti^4+^ site). In K-edge X-ray spectroscopy data confirmed In was uniformly present in the In^3+^ oxidation state, yet the prevailing In coordination environment was consistent with accommodation in the Ti^4+^ site in sixfold coordination, contrary to the targeted formulation. Nevertheless, single phase zirconolite-2M was able to form in the Ca_1-x_Zr_1-x_In_2x_Ti_2_O_7_ system for 0.10 ≤ x ≤ 0.20. Progressive substitution of In^3+^ beyond x ≥ 0.20 promoted the formation of several In-titanate phases, namely In_2_TiO_5_ and InTi_0.75_Ca_0.25_O_3.25_, alongside TiO_2_ and c-ZrO_2_. In^3+^ was also utilised to successfully charge compensate U^4+^ and U^5+^ resulting in the formation of near single phase zirconolite-2M (accompanied by ~ 1–2 wt% ZrO_2_) in the Ca_1-x_U_x_ZrTi_2-2x_In_2x_O_7_ system at x = 0.05 and 0.10. The dominant oxidation states of U^4+^ (argon synthesis) and U^5+^ (air synthesis) were determined by U L_3_-edge XANES analysis. Analysis of the XANES and EXAFS region of the Zr K-edge in these materials was consistent with Zr^4+^ occupying sevenfold coordinated sites in the zirconolite structure, as would be expected. These data form a useful contribution towards ongoing efforts to design, characterise and performance test titanate wasteform materials substituted with appropriate quantities of neutron poisoning additives.

## Supplementary Information

Supplementary Information.

## Data Availability

The datasets used and/or analysed during the current study available from the corresponding author on reasonable request.
