# Supplementary material for: Underpinning the use of indium as a neutron absorbing additive in zirconolite by X-ray absorption spectroscopy
Source: Sci Rep. 2023 Jun 8;13:9329. doi: 10.1038/s41598-023-34619-5 (PMC10250489; doi:10.1038/s41598-023-34619-5)

**Supplementary Information: Underpinning the Use of Indium as a Neutron Absorbing Additive in Zirconolite by X-ray Absorption Spectroscopy**

Lewis R. Blackburn^a^, Luke T. Townsend^a^, Malin C. Dixon Wilkins^a^, Toshiaki Ina^b^, Merve Kuman^a^,
Shi-Kuan Sun^a,c^, Amber R. Mason^a^, Laura J. Gardner^a^, Martin C. Stennett^a^, Claire L. Corkhill^a^
and Neil Hyatt^a^

^a^ Immobilisation Science Laboratory (ISL), University of Sheffield, Department of Materials Science and Engineering, Sir Robert Hadfield Building, Mappin Street, S13JD, UK

^b^ Spring8 (JASRI), 1-1-1 Kouto, Sayo-cho, Sayo-gun, Hyogo 679-5198, Japan

^c^ School of Materials Science and Energy Engineering, Foshan University, Foshan 528000, China

Corresponding author – [lewis.blackburn@sheffield.ac.uk](mailto:lewis.blackburn@sheffield.ac.uk)

**
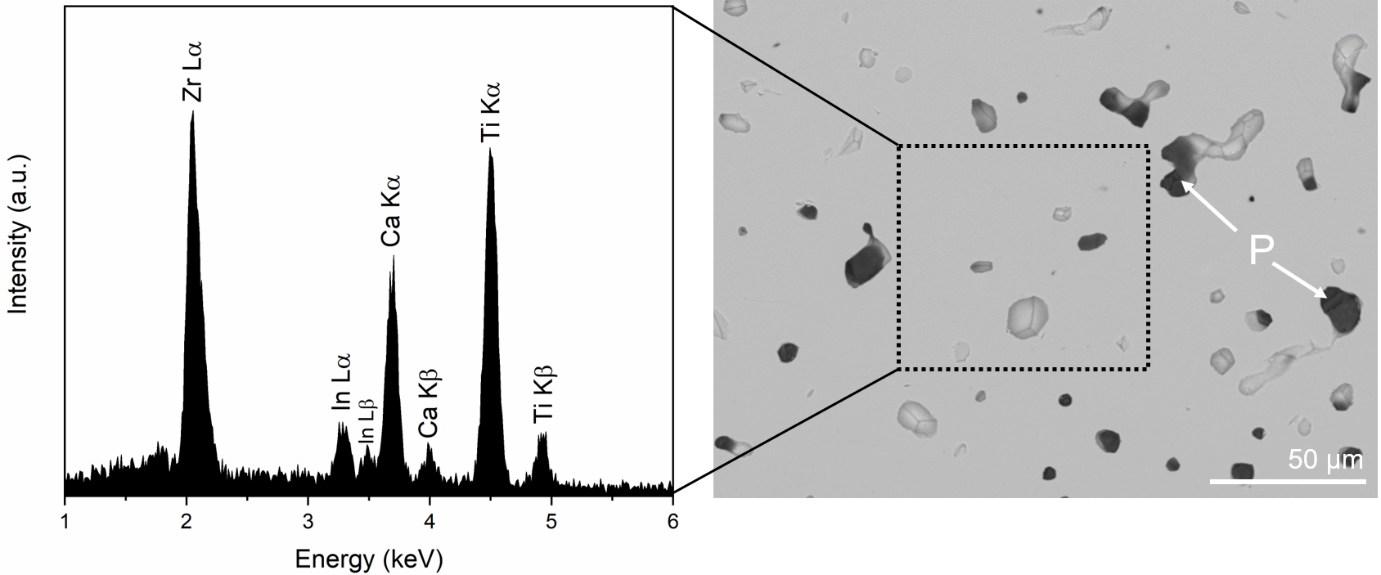
**

**Fig. S1**) EDS spectrum for the x = 0.10 composition in the Ca_1-x_Zr_1-x_In­_2x_Ti_2_O_7_ solid solution. Areas marked P are labelled as porosity.

**Table S1**) Approximate EDS compositions for the x = 0.10 and 0.20 compositions in the
Ca_1-x_Zr_1-x_In­_2x_Ti_2_O_7_ solid solution

| **Nominal Composition** | **Average Composition from EDS** |
| --- | --- |
| x = 0.10 | Ca_0.91(3)_Zr_0.82(2)_In_0.17(1)_Ti_2.10(3)_O_7_ |
| x = 0.20 | Ca_0.79(2)_Zr_0.73(3)_In_0.35(2)_Ti_2.13(1)_O_7_ |

**Table S2**) Fitting parameters for Zr K-edge EXAFS data presented in **Fig. 10**


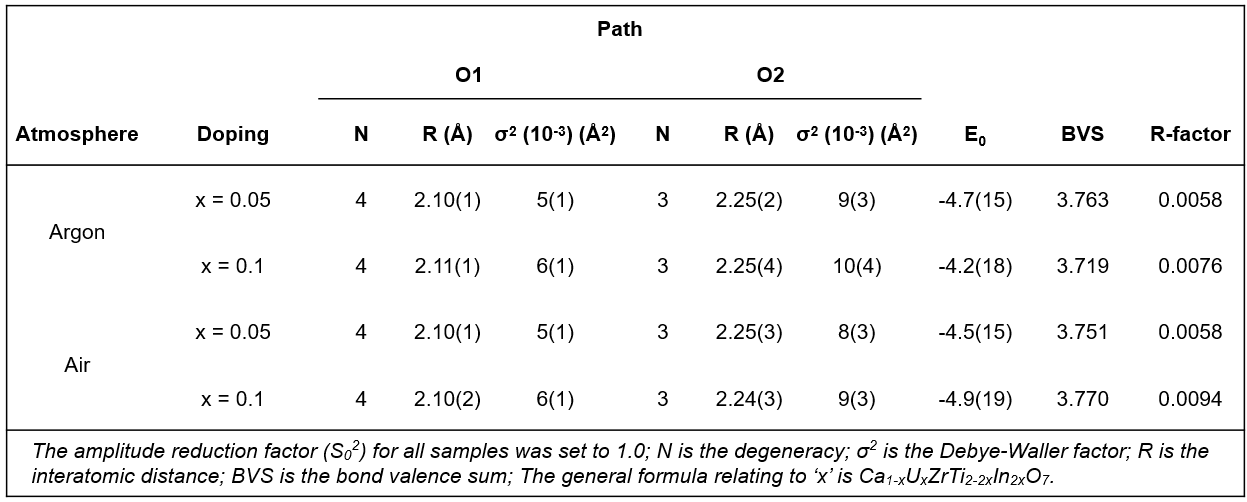

Supplement: Supplementary file 1 — Supplementary Information. [file 41598_2023_34619_MOESM1_ESM.docx]
